# Value-Based Health Care Implementation: The Case Study of mTBI Biomarkers

**DOI:** 10.3390/jpm14060634

**Published:** 2024-06-14

**Authors:** Martina Zibetti, Chiara Di Resta, Giuseppe Banfi, Rossella Tomaiuolo

**Affiliations:** 1 Faculty of Medicine, Università Vita-Salute San Raffaele, 20132 Milan, Italy; 2 IRCCS Galeazzi-Sant’Ambrogio, 20157 Milan, Italy

**Keywords:** traumatic brain injury (TBI), sports-related TBI, brain injury markers/biomarkers, TBI biomarker, value-based health care implementation (VBHC)

## Abstract

Traumatic brain injury is a significant global health issue, affecting approximately 69 million people annually. Early diagnosis is crucial for effective management, and biomarkers provide a promising approach to identifying traumatic brain injury in various settings. This study investigates the perceived usefulness of biomarker testing in two distinct contexts: emergency departments and sports settings. Comprehensive interviews were conducted among healthcare professionals in emergency departments and sports-related medical staff. The interviews assessed their perceptions of the diagnostic accuracy, practicality, and overall value of traumatic brain injury biomarker testing. The findings indicate that the perceived usefulness of biomarker testing is high among professionals in both settings. However, significant differences emerged in the perceived barriers to implementation, with emergency department staff citing logistical issues and sports professionals expressing cost concerns. Addressing identified barriers could enhance the adoption and effectiveness of these tests, ultimately improving patient outcomes. Future research should focus on optimizing testing protocols and reducing implementation challenges. This study aims to evaluate the implementation of mild traumatic brain injury biomarkers within the framework of value-based health care, focusing on diagnostic accuracy and patient outcomes.

## 1. Introduction

Technological innovation in diagnostic testing presents an opportunity for all healthcare service providers—from healthcare professionals and laboratory specialists to device manufacturers—to explore innovative solutions, gain new insights into their applications in specific contexts, and generate a virtuous adoption cycle. This collaborative process ensures appropriate use and clinical stewardship of laboratory data [1].

The appropriateness of care is central to creating value. Assessing this value requires measuring clinical outcomes such as patient health improvements, life expectancy, functionality, and quality of life.

The clinical stewardship of laboratory data involves the responsible management, interpretation, and utilization of laboratory test results to optimize patient care and improve healthcare outcomes. This concept integrates principles of value-based medicine, quality improvement, and interprofessional collaboration.

Value-based health care (VBHC) is a strategic trend that guides healthcare innovation by emphasizing the evaluation of outcomes rather than just technological features, fostering collaboration instead of isolation, and promoting partnerships with device manufacturers rather than purely transactional relationships [1]. 

Translating these theoretical concepts into concrete actions requires precise alignment among the various stakeholders in the healthcare ecosystem. This strategy emphasizes integrating evidence-based practices, quality assurance, and interprofessional collaboration to enhance the efficiency and effectiveness of healthcare services. 

In laboratory data management, ensuring appropriate test utilization is paramount to avoid unnecessary and redundant testing. This not only streamlines the diagnostic process but also conserves medical resources.

Collaboration between laboratory professionals, laboratory test developers, and other healthcare providers is essential for effectively using laboratory data. This collaboration is supported by feedback mechanisms that allow clinicians to provide input on the utility and clarity of laboratory reports, fostering a culture of continuous improvement.

This study primarily aims to evaluate the implementation of mild traumatic brain injury (mTBI) biomarkers within the framework of value-based health care, with a specific focus on diagnostic accuracy and patient outcomes.

### The Case Study of Traumatic Brain Injury Biomarkers

Every year, traumatic brain injury (TBI) affects an estimated 69 million individuals worldwide [2]. The World Health Organization (WHO) Neurotrauma Task Force defines TBI as a blow to the head resulting in acute brain function impairment. Certain demographic groups are particularly susceptible: adults over 75, children under 5, and adolescents between 15 and 24 [3]. TBI has significant public health and socioeconomic impacts due to its high rates of mortality, morbidity, and disability [2]. 

The Glasgow Coma Scale (GCS) is the standard tool used in emergency rooms (ER) to classify TBI severity into three categories: severe (GCS 3–8), moderate (GCS 9–13), and mild (GCS 14–15). These categories help assess the risk of intracranial hematomas, the potential need for neurosurgery, and the likely prognosis [4].

Computed axial tomography (CT) is the most used method for radiological examination [5], with an estimated sensitivity of 100%, a positive predictive value of 10%, a negative predictive value of 100%, and a specificity of 51% [6]. While magnetic resonance imaging (MRI) is more sensitive for detecting intracranial hemorrhages and edema [7], CT scans remain the first choice in emergency settings [8]. However, performing CT scans for all patients is undesirable due to the limited prevalence of positivity, radiological risks, prolonged ER stays, resource inefficiencies, and high cost and complexity [5].

mTBI constitutes 90% of all brain injuries [9], with the primary symptoms being a brief loss of consciousness (less than 30 min), confusion, or post-traumatic amnesia not attributable to other factors like psychological trauma or alcohol/drug intoxication [3]. mTBIs are about 15 times more frequent than moderate TBIs and over 20 times more frequent than severe TBIs [4]. It is prevalent among athletes in collision sports and military personnel but also results from falls, vehicle accidents, and assaults [10]. 

The Mild TBI Committee of the American Congress of Rehabilitation Medicine, revised by the WHO, defines mTBI as a TBI with a Glasgow Coma Scale score between 13 and 15 at 30 min post-injury, with one or more of the following symptoms: <30 min loss of consciousness; <24 h post-traumatic amnesia (PTA); impaired mental state at the time of the accident; and/or transient neurological deficit [11].

It is crucial to consider that mTBI can sometimes be associated with severe intracranial injuries, posing risks of complications, particularly intracranial bleeding, both immediately after the trauma and after months. Risk stratification should incorporate clinical history, trauma dynamics, and medication data [12]. 

Additionally, mTBIs can contribute to neurodegenerative disorders and long-term behavioral and neurological impairments [13]. Repeated mTBIs may also lead to progressive neurological conditions, necessitating the identification of at-risk individuals to prevent premature returns to play or duty while the brain remains vulnerable [10,14]. In addition to primary trauma, secondary injuries can trigger a cascade of cellular and molecular reactions that may result in neuronal and astroglia injuries, axonal damage, and inflammation [13].

Overall, given the complexity and potential risks associated with concussion management, including the possibility of long-term neurological impacts, the development and use of biochemical biomarkers are crucial for improving the accuracy of diagnosis and guiding more effective treatment strategies. Fluid biomarkers offer a promising avenue for developing predictive models to improve risk stratification and support clinical decision-making [10,14].

## 2. Materials and Methods

To effectively investigate the introduction of TBI biomarkers in the mTBI diagnostic process, we explored (1) the context analysis and (2) the diagnostic setting characterization. 

### 2.1. Context Analysis

The context analysis focused on TBI guidelines and markers (typology and methods).

A comparison of guidelines for TBI patient management was carried out, and a literature review was conducted focusing on biomarkers released after TBI and diagnostic instruments available for their detection. The consulted guidelines were the “Canadian CT Head Rule”, the “Scandinavian Guidelines”, the “New Orleans Criteria for TC scan in mild head injury”, and the “guidelines of the National Institute for Health and Care Excellence” (NICE 2014), and those of the Neurotraumatology Committee of the World Federation of Neurosurgical Societies (NCWFS). 

The search for bibliographic sources regarding TBI biomarkers was carried out on PubMed, Science Direct, and Cochrane Library for indexed articles in English. The following keywords were used: “brain injury markers”, “brain injury biomarkers”, “neurodegeneration biomarkers”, “TBI biomarker”, and “TBI markers”. The bibliographic sources for the analysis of the methodological assessment of TBI biomarkers and the technological development stage of biomarker detection devices were found by searching PubMed with the following keywords: “methods detection TBI biomarkers”, “TBI biomarkers detection devices”, “electrochemical sensors TBI”, “TBI biomarkers measurement methods”, and “optical detection TBI”. The medical subject headings (MeSH) terms used for our PubMed search included brain injury markers, brain injury biomarkers, neurodegeneration biomarkers, TBI biomarkers, and TBI markers.

The temporal window for all the research was fixed between January 2012 and December 2023 to focus on the most recent publications. Manual searches for older but significant references cited in the reviewed articles were made when appropriate. All the articles included in this literature review were published in journals with an impact factor higher than 4. This criterion was selected to ensure the inclusion of high-quality studies with substantial impact in the field. While we acknowledge that many reputable journals have impact factors in the range of 3 to 4, we intended to prioritize the most influential research.

### 2.2. Diagnostic Setting Characterization 

A strategic, multi-faceted methodology was employed to thoroughly assess the potential diagnostic setting for TBI biomarkers, combining SWOT (strengths, weaknesses, opportunities, and threats) analysis, stakeholder matrix, stakeholder interviews, and patient journey mapping. 

First, a SWOT analysis was conducted to examine the strengths, weaknesses, opportunities, and threats associated with the potential of implementing mTBI biomarkers in emergency rooms. 

Next, a stakeholder matrix was created to identify critical stakeholders with significant interests and decision-making authority. A stakeholder matrix is a tool used to identify individuals, groups, or organizations involved in a project or who can influence, be influenced by, or perceive themselves as affected by the project’s decisions, activities, or outcomes. Stakeholders are classified based on their power and interest levels. Those with high power and high interest are managed closely, while those with high power but low interest are kept satisfied. Stakeholders with low power but high interest should know the project’s progress. Finally, those with low power and interest are monitored with minimal effort to see if their attitudes shift over time. This categorization helps ensure appropriate engagement and communication with each stakeholder throughout the project. 

Three different stakeholder matrixes were made considering three different situations/ways/methods of implementation for the biomarkers: (1) inclination to use laboratory exams for traumatic brain injury biomarkers, (2) inclination to introduce point-of-care testing (POCT) devices for traumatic brain injury biomarkers in the ER, and (3) inclination to introduce POCT devices for traumatic brain injury biomarkers directly on-site. Therefore, the identified stakeholders were then interviewed to determine their disposition toward using traumatic brain injury biomarkers.

Finally, based on interview responses, current patient journeys were analyzed and implemented patient journeys were defined.

## 3. Results

### 3.1. Context Analysis

The current ER guidelines analysis revealed that uniformity in guidelines for managing traumatic brain injury is lacking nationally and internationally. Diagnostic, admission, and discharge decision policies vary among centers in emergency departments and hospital wards [15]. Guidelines commonly focus on assessing the patient’s mental state, cranial nerves, sensory awareness, motor functions, and reflexes. Patients receiving antiplatelet/anticoagulant therapy should stop the treatment during observation [15]. 

Neurological imaging, particularly computed tomography, is the primary method for identifying a head injury [5]. However, each country has its directives for CT use. For example, CT for minor head injury cases is used selectively in Europe and Canada; in Italy, CT is only recommended if a fracture is visible on skull radiography; in Denmark, it is rarely ordered, and then it can be prescribed only by a neurosurgeon; in the UK and Spain, CT is only recommended for cases with a documented skull fracture, focal neurological deficit, or deterioration in the mental state [16].

The most frequently used guidelines include the Canadian CT Head Rule, the Scandinavian Guidelines, the New Orleans Criteria for CT scan in mild head injury, the National Institute for Health and Care Excellence (NICE 2014) guidelines, and those of the Neurotraumatology Committee of the World Federation of Neurosurgical Societies (NCWFS) [15]. These guidelines differ in terms of the parameters considered: the Canadian CT rules are based on five high-risk and two medium-risk criteria [16]; the Scandinavian guidelines consider the serum levels of S100 calcium-binding protein B (S100B) [17,18]; the New Orleans Criteria for CT scan (NOC) includes seven items and was only developed for use in patients with a GCS score of 15 [19]; the NICE 2014 guidelines are based upon the Canadian CT Head Rule and lead to more CT scans being performed, but fewer skull radiographs and admissions [20,21]; lastly, the NCWFS protocol is similar to the NICE guidelines but is less strict and leads to more CTs [22,23].

Moreover, in sports contexts, TBIs are often referred to as sports-related concussions (SRC). These concussions are caused by biomechanical forces and are characterized by several distinctive features. Typically, they result in a rapid onset of short-lived neurological impairment that resolves independently, although symptoms can sometimes evolve over several minutes to hours. They are caused by a direct impact on the head, face, or neck or by an effect elsewhere on the body that transmits an impulsive force to the head.

SRCs can lead to neuropathological changes, but their acute clinical signs generally reflect functional disturbances rather than structural injuries, which means standard neuroimaging often does not detect abnormalities. The range of symptoms includes neurological impairment that may or may not involve loss of consciousness and cannot be attributed to drugs, alcohol, medications, other injuries, or comorbidities like psychological factors or pre-existing medical conditions.

The resolution of clinical and cognitive symptoms typically follows a sequential course, although in some cases, these symptoms can be prolonged. Most SRCs occur without loss of consciousness or clear neurological signs, and there remains no definitive diagnostic test or marker for immediate diagnosis in sports settings. If a concussion is suspected, the athlete should be removed from the playing field and assessed by a licensed healthcare provider [24].

SRC is one of the most challenging injuries to diagnose, assess, and manage in sports medicine, underscoring the importance of a prompt and accurate diagnosis. Current diagnostic approaches rely on evaluating somatic, cognitive, and emotional symptoms and physical signs like loss of balance or consciousness. Still, there are no universal guidelines for SRC management, with each sports federation offering its recommendations (for instance, the Scottish FA Heading Guidelines, the World Rugby Concussion Guidelines, etc.) for SRC diagnosis, exemption from play, and gradual resume of training after the injury. 

The 2017 Concussion in Sport Group (CISG) consensus statement aimed to improve the conceptual understanding of SRC. Using an expert consensus-based approach, they developed the Sports Concussion Assessment Tool version 5 (SCAT5). This tool provides a standardized sideline assessment that helps distinguish concussed from non-concussed athletes. When a concussion is suspected, the athlete should be immediately removed and evaluated using SCAT5 [24]. 

The SCAT5 evaluates sports-related concussions through five key phases [25]. First, it involves a red flags assessment, an initial screening where healthcare professionals identify urgent danger signs such as severe neck pain, worsening headache, or repeated vomiting. If any of these “red flags” or observable signs are noted following a direct or indirect blow to the head, the athlete is immediately and safely removed from play for evaluation by a licensed healthcare provider. Next is the observable signs assessment, where professionals visually check for lying motionless on the field, difficulties with balance or motor coordination, confusion, or a blank/vacant look. Facial injuries after head trauma are also noted. In the memory assessment phase, a series of questions are asked to gauge the athlete’s memory, determining if the concussion has affected their ability to recall recent events. The GCS is used to assess the level of consciousness by evaluating verbal, motor, and eye-opening responses, helping identify the severity of the concussion. Finally, the cervical spine assessment phase evaluates neck pain, range of motion, and sensation in the neck region to ensure that the cervical spine is undamaged. Strength and stability are also assessed.

The SCAT5 thus provides a thorough, structured approach to assess sports-related concussions, allowing healthcare professionals to make informed decisions on athlete management and return-to-play protocols.

Return to training/play should commence only after symptoms have completely disappeared and should follow a stepwise approach. Table 1 shows a graduated return-to-sport (RTS) strategy, according to the 2017 Concussion in Sport Group consensus statement. Before initiating the RTS progression, an initial 24–48 h of relative physical and cognitive rest are strongly recommended. If symptoms worsen during exercise, the athlete should revert to the previous step for further recovery [24]. 

Regarding the analytical methods for mTBI biomarkers, the ideal device would be portable and usable by untrained personnel in non-ideal conditions (e.g., extreme conditions of temperature and humidity), requiring a small sample volume and a minimal sample preparation (ideally a drop of whole capillary blood obtained via a fingerstick) to quickly measure a panel of biomarkers with high sensitivity and specificity simultaneously. The instrument’s lower detection limit must be below the cut-off concentration to distinguish the TBI from the physiological situation. Also, reagents should be stable, and manufacturing should be inexpensive to mass-produce devices [26,27]. 

To date, the TBI plasma cartridge for the Abbott i-STAT Alinity is the most appropriate for introduction in mTBI patient management. Abbott’s test can be run on the Alinity i system (a panel of in vitro diagnostic chemiluminescent microparticle immunoassays (CMIA) that are performed in a clinical laboratory) or on the i-STAT Alinity platform, which is a portable device that uses electrochemical detection (amperometry) to measure GFAP and UCH-L1 levels in multiplexing in an EDTA anticoagulated plasma sample of 20 µL in 15 min. We focused our analysis on the i-STAT Alinity platform. The device is designed to be used by trained personnel in a clinical laboratory setting, and a positive result identifies patients who need a CT scan [28]. It was approved by the FDA in January 2021 and received the CE Mark in December 2021, and it is currently available outside the U.S. For now, the i-STAT Alinity TBI cartridge requires an EDTA anticoagulated plasma sample, which healthcare professionals prepare in a clinical laboratory setting. Implementing a test that works with a whole blood sample would allow the use in field settings since the device is already portable [27,29,30].

Based on the evidence, the Abbott test has great potential for improving traumatic brain injury trauma management since it exploits two suitable and reliable biomarkers and is already adequate for commercialization [27].

Not only the chosen biomarkers but also the modality of test execution can impact the effectiveness of the test in everyday practice. Hence, we identified three different settings for testing: the ER, with standard lab methods; the ER, with a POCT; and on-site testing, with a POCT.

### 3.2. Characterization of the Diagnostic Setting

**Figure 1:**
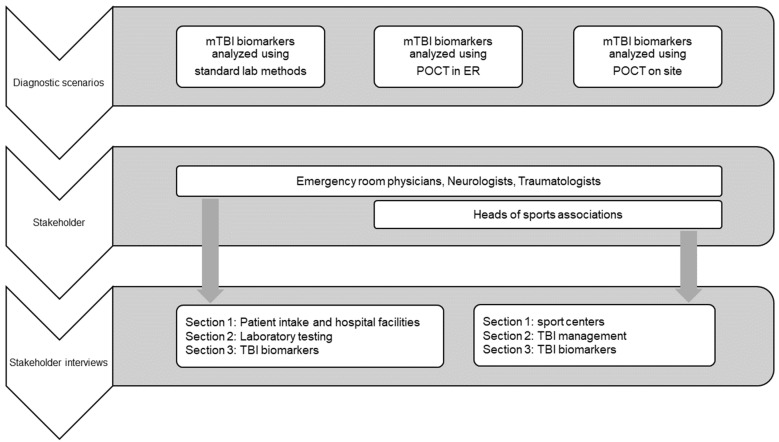
Schematization of the characterization of the different diagnostic settings.

In this section, the SWOT and stakeholder analysis results are reported, along with an elaboration of the answers to the interviews from head ER physicians and heads of sports associations, as schematized in Figure 1.

**Figure 2:**
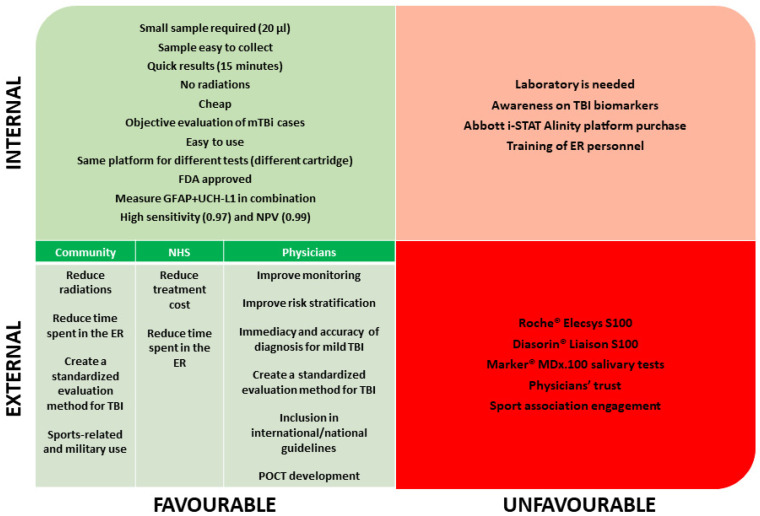
The graph summarizes a SWOT analysis for implementing biomarker testing in managing traumatic brain injury.

Results from the SWOT analysis are summarized in Figure 2. The SWOT analysis provides a strategic framework to evaluate the possibility of implementing Abbott i-STAT Alinity in emergency rooms. It offers a fact-based assessment of internal and external factors influencing its adoption. Strengths and weaknesses are internal, controllable factors: strengths provide competitive advantages and help leverage opportunities or counter threats, while weaknesses represent vulnerabilities that hinder competitiveness. Opportunities and threats are external, uncontrollable factors: opportunities offer favorable conditions for competitive advantages, while threats pose potential risks to the organization’s success. 

The strengths are that the Abbott i-STAT Alinity works with a very small blood sample (20 μL) and gives quick results (15 min). Compared to the CT scan, the test’s execution does not require ionizing radiations, and its cost is lower than that of a CT scan. The test allows for the assessment of objectively mild TBI cases by measuring GFAP and UCH-L1 in combination, which has shown the highest accuracy in terms of the area under the curve (AUC) for discriminating between mTBI patients and healthy controls. The Abbott i-STAT Alinity platform is straightforward to use, and the same platform can be used for many different tests by just changing the cartridge. The test has a sensitivity of 0.97 (95% CI) and an NPV of 0.99 (95% CI), with <1% of patients reporting a positive result in a CT scan when the test was negative (data from the multicenter trial ALERT-TBI 2012–2014), and the FDA already approves it.

The main weakness of the i-STAT Alinity TBI plasma test is that, for now, it is not a portable test since it needs a laboratory to centrifuge the sample. Also, to introduce the test in the ER routinely, the ER needs to purchase the Abbott i-STAT Alinity platform and cartridges if they are not already used in the ER, and the medical staff (primarily nurses) must learn how to use a new instrument. To date, there is a low awareness about biomarkers for TBI among physicians, patients, and caregivers, and this is an issue that must be amended to introduce the use of biomarkers in ER routines.

There are many opportunities for the community: using biomarkers can reduce exposure to radiation by reducing the number of performed CT scans; it can also reduce the permanence time in the ER, reducing, therefore, ER overcrowding. Biomarker tests can help create a standardized evaluation method for TBI by including biomarkers in international guidelines, which is most helpful in sports-related and military contexts. From the point of view of the National Health System (NHS), the test enables the opportunity to reduce treatment costs by performing fewer CT scans and reducing the permanence time in the ER, therefore reducing ER overcrowding. Lastly, from physicians’ perspectives, the test improves TBI patients’ monitoring, improves TBI patients’ risk stratification, and enables immediacy and accuracy of diagnosis for mild TBI through a standardized evaluation method. The further implementation as POCT in the future and the inclusion in national/international guidelines are also significant opportunities for physicians.

The threats are represented by possible competitors, such as the Roche^®^ Elecsys S100 test (Roche, Germany) [31], the Diasorin^®^ Liaison S100 test (Diasorin, Italy) [31], and the Marker^®^ MDx.100 Concussion/mTBI Diagnostic salivary tests [32]. However, these tests are based on different biomarkers with lower specificity and accuracy than GFAP and UCH-L1; therefore, their potential use differs. Another threat is that medical staff’s trust in biomarkers is not granted since many may continue to consider a CT scan more reliable than biomarkers. Lastly, the engagement of sports associations, which is fundamental to allowing the introduction of the test in the sports context, is not to be taken for granted.

Three distinct diagnostic scenarios to analyze TBI biomarkers were derived from the SWOT analysis considerations: (1) standard laboratory methods; (2) POCT devices in the emergency room; and (3) POCT devices used directly on site.

**Figure 3:**
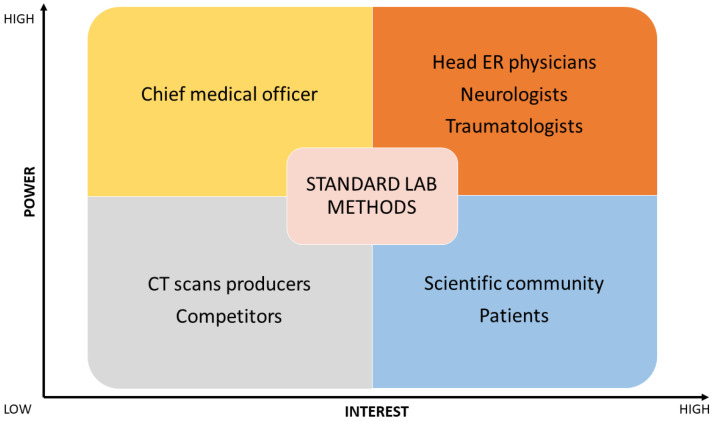
Stakeholder matrix for introducing biomarker testing as a standard lab method. ER, emergency room; CT, computed tomography.

**Figure 4:**
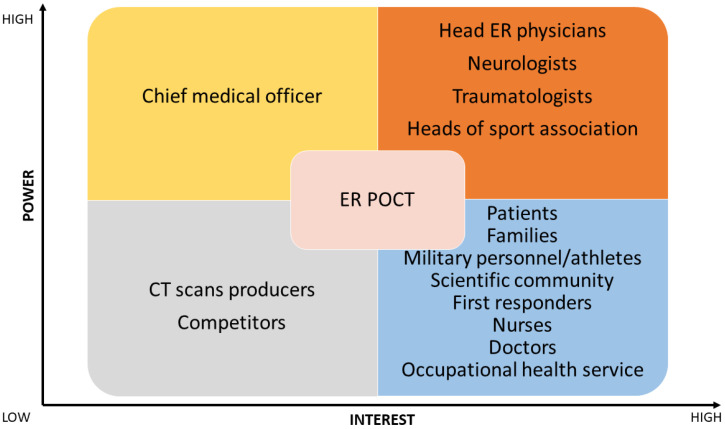
Stakeholder matrix for introducing biomarker testing as a point-of-care testing (POCT) in emergency rooms (ER). CT, computed tomography.

**Figure 5:**
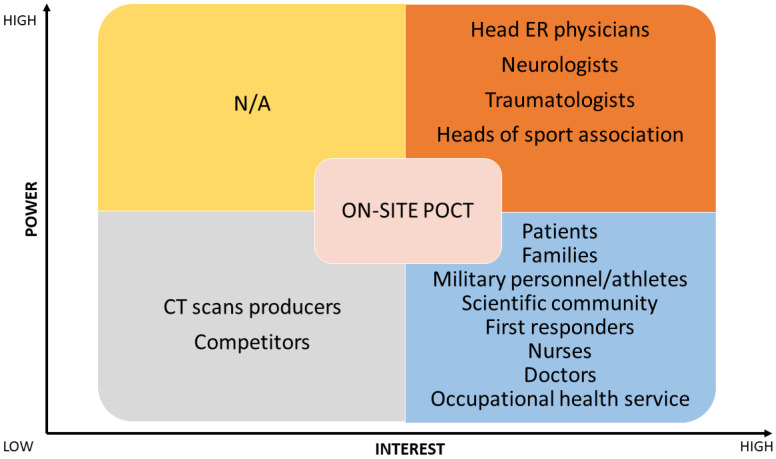
Stakeholder matrix for introducing testing as a point-of-care testing (POCT) on-site. ER, emergency room; CT, computed tomography.

In the first scenario (see Figure 3), the matrix highlighted head ER physicians, neurologists, and traumatologists as stakeholders. In the second (see Figure 4) and third scenarios (see Figure 5), head ER physicians, neurologists, traumatologists, and heads of sports associations were highlighted as stakeholders.

The heads of ERs were interviewed directly to outline the current patient journey of mTBI patients in Italy and to gauge their perception of the utility and practicality of mTBI biomarker testing in the ER setting. The interviews aimed to gather information on the following areas: procedures for managing suspected TBI cases, ER organization (including CT scan and clinical lab availability and personnel training), standard laboratory tests, and the willingness to adopt predictive biomarkers. It was structured into three sections covering details about the medical center and patient management (15 questions), laboratory tests (16 questions), and TBI biomarkers (30 questions). The questions ranged from yes/no answers to multiple-choice and open-ended formats, and the interview was administered to nine head ER physicians across various Italian hospitals (in the Lombardy Region).

#### 3.2.1. Interview for Heads of ER

The most significant data drawn from the processing of the responses to the questionnaires were summarized; the full data can be consulted in the appendix.

The results from the first section of the interview (questions 1–15, Supplementary Materials), focusing on patient intake and hospital facilities, reveal critical aspects of the typical patient journey for suspected TBI cases in ERs. Half of the centers report a common sequence where a patient arrives at the ER, undergoes triage, meets with an ER physician who determines the necessity of a CT scan, and, based on the scan results, may undergo further laboratory tests before a specialist consultation leads to either discharge or hospitalization. 

**Figure 6:**
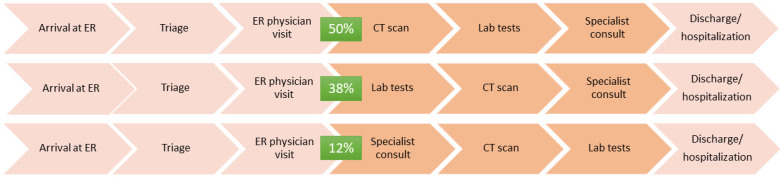
Current TBI patient journeys.

In 37.5% of the centers, laboratory tests are conducted before the CT scan, while in 12.5% of the cases, a specialist is consulted before any scans or tests (see Figure 6). The initial assessment and subsequent categorization of patients into risk levels are predominantly managed by ER nurses, who evaluate factors like the GCS score, anticoagulant therapy, age, dynamics of trauma, and other risk factors to classify patients as low-, intermediate-, or high-risk. Three distinct levels are identified in categorizing TBI patients based on risk, each defined by symptoms and factors assessed primarily by the attending ER nurses. Patients classified as low-risk exhibit full orientation with a GCS score of 15, indicating no significant risk factors linked to the trauma they have experienced. This suggests their condition is stable and may require less intensive emergency care.

Those falling into the intermediate-risk category may not have significant risk factors, but they present with symptoms that require cautious monitoring. These symptoms can include retrograde amnesia, dynamics of the trauma that are considered high-risk, vomiting, indications of substance abuse, coagulopathy, severe headaches, or a known history of epilepsy. Each of these factors complicates their clinical picture and necessitates a thorough evaluation to determine the appropriate course of treatment.

Lastly, the high-risk category comprises patients who, despite having a GCS of 15 and perhaps a brief loss of consciousness, exhibit additional significant risk factors such as advanced age, evidence of substance intoxication, or specific conditions like coagulopathy. This group also includes those experiencing a comitial post-traumatic crisis or who have a GCS score of 14. For these patients, the risks are more pronounced and potentially life-threatening, demanding immediate and aggressive management to mitigate severe complications.

Further findings indicate that 88.9% of the interviewed centers have immediate ER CT scanner access, typically available within 20 min. However, waiting times can extend to two hours in 22.2% of cases. Post-evaluation, nearly two-thirds of the centers require a mandatory six-hour observation period for all TBI patients, regardless of the CT scan results. This standard procedure places a significant resource burden on the ER.

All interviewed physicians unanimously see the value in potentially eliminating the need for a CT scan upon arrival, which could significantly streamline the process and reduce the resource load in emergency settings. This insight underscores the importance of developing and integrating rapid diagnostic tools like TBI biomarkers, which could facilitate immediate and effective decision-making at the point of care.

The interview results from Section S2 related to laboratory testing (questions 16–31, Supplementary Materials) reveal a comprehensive view of laboratory testing practices in ERs concerning TBIs. Almost all ERs (88.8%) centralize their laboratory services, while a smaller proportion (11.1%) dedicate their labs exclusively to emergencies. These laboratories operate around the clock, and most ER physicians (88.9%) report that their requests are processed efficiently.

**Figure 7:**
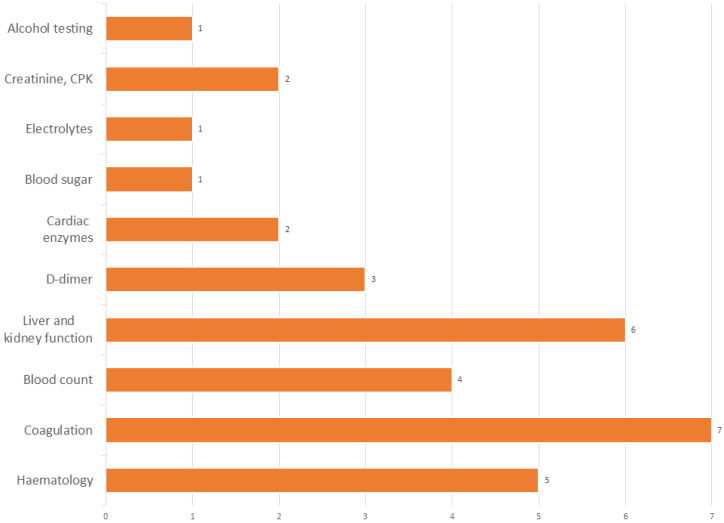
Laboratory exams are usually requested in case of TBIs. The chart highlights commonly requested tests, like blood counts, coagulation, hematology, and liver/kidney function, showing the consistent use of lab exams in TBI assessment and the multifaceted approach needed in emergency care.

Regarding TBI management, slightly more than half of the physicians (55.6%) routinely request laboratory tests to support the diagnosis (Figure 7). The tests most frequently utilized include blood counts, coagulation studies, and liver/kidney function tests. The variety of tests gives a comprehensive view of the patient’s condition, helping to identify other complicating factors that might affect TBI management. Figure 7 emphasizes the varied but consistent use of laboratory exams in the diagnostic and prognostic assessment of TBIs, indicating the multifaceted approach often required in emergency settings.

However, 44.6% feel these tests do not provide additional value beyond what can be gleaned from CT scans, nor do they enhance the diagnostic pathway. The specific laboratory tests requested are detailed in Figure 7, indicating the proportion of centers that utilize each test. For diagnosing TBIs, half of the physicians use these tests solely for diagnostic purposes, a quarter for prognostic purposes, and the remainder for diagnostic and prognostic reasons.

The turnaround time for test results varies, with 22.2% of ERs reporting a 30 min wait, 33.3% a one-hour wait, 22.2% a two-hour wait, and 11.1% reporting up to four hours. Despite the varied testing practices, physicians unanimously agree that laboratory tests do not significantly reduce the number of CT scans performed.

Section S3 delves into the awareness and use of TBI biomarkers among ER physicians (questions 32–61, Supplementary Materials). While only 55.6% of physicians are familiar with TBI biomarkers, a slightly lower percentage (44%) specifically know about GFAP and UCH-L1. Despite this, there is a strong consensus (100%) on the potential benefits of integrating biomarker testing into ER protocols, particularly for its diagnostic accuracy and speed. Most physicians (66.7%) prefer POCT for its quickness and ease of use, though a third would opt for traditional lab tests to avoid additional staffing needs. Some physicians (22.2%) also suggest a follow-up lab test to confirm POCT results when positive.

**Figure 8:**
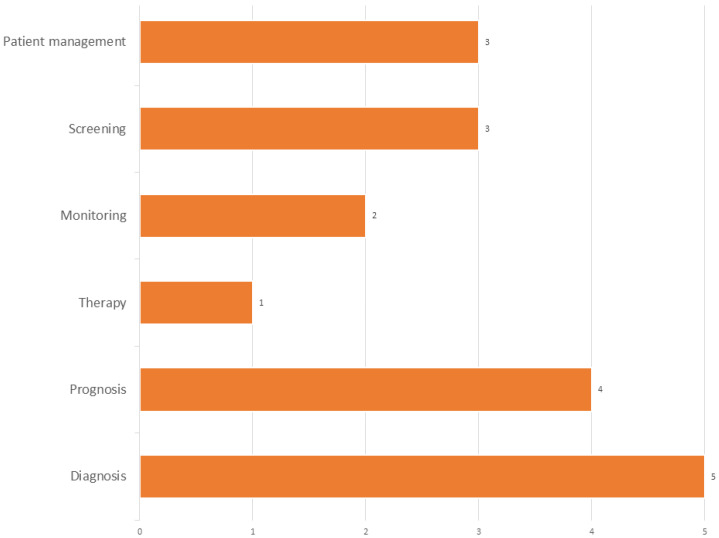
The purpose of including biomarker testing in the ER routine. The graph illustrates the potential benefits of integrating biomarker testing into emergency care routines for TBIs, including better diagnosis (28%), prognosis (22%), patient management, and screening (both at 17%).

The intended uses for biomarker testing are varied, as depicted in Figure 8. The primary application favored by physicians is for diagnostic purposes. 

**Figure 9:**
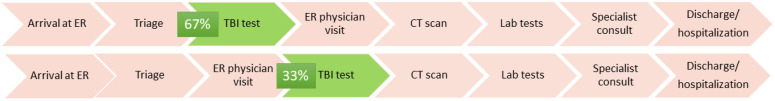
Patient journey for TBI patients using biomarker testing. The figure highlights the pivotal role of POCT in reducing the reliance on CT scans, thereby expediting the diagnosis and treatment of TBI patients, which could lead to better outcomes and more efficient use of medical resources.

Physicians are divided on when to introduce biomarker testing, with 66.7% favoring its use at triage and 33.3% after admission. The envisaged new patient journey incorporating biomarker testing is illustrated in Figure 9. 

Key conditions for integrating POCT into routine ER operations include the availability of lab equipment (41.7%), trained staff (25%), and ensuring high diagnostic accuracy (33.3%). A total of 85.7% of the physicians express confidence in a validated POCT replacing CT scans. Currently, 77.7% of ERs use different POCTs, underscoring the current adoption and utilization of these rapid diagnostic tools in ER settings; the future could see POCTs being used directly at the site of injury, with most physicians (66.7%) agreeing that repeat lab tests in the ER would be unnecessary in such cases.

#### 3.2.2. Interview for Heads of Sports Associations

Federal physicians and heads of sports associations were interviewed to evaluate the potential application of mTBI biomarkers in the sports context, offering insights into how these biomarkers could be used outside the ER. The interview for the heads of sports associations sought data on sports practices and facilities, management of suspected TBIs, and the willingness to use predictive biomarkers. This interview was divided into three sections covering information about the sports center (one open-ended question), TBI management (fourteen questions), and tests and biomarkers for TBI (nine yes/no questions). The interview involved eight heads of sports associations and federal physicians across disciplines like rugby, boxing, cycling, motorcycling, and sailing. The interview responses from heads of sports associations highlight the diverse approaches and requirements for managing TBIs across different sports, reflecting each discipline’s unique challenges and environments. For this reason, the results of Sections S1 and S2 (Supplementary Material) of the interview (questions related to the sports center and related to trauma management) are summarized and reported here and divided by sport in Table 2.

These insights demonstrate a tailored approach to TBI management across sports, emphasizing the necessity of sport-specific protocols and the potential benefits of integrating innovative diagnostic tools like POCTs to enhance safety and care.

The interview responses revealed a general lack of awareness among heads of sports associations regarding TBI biomarkers, with only 50% familiar with any specific biomarkers, including GFAP and UCH-L1. None knew the diagnostic window for these biomarkers. However, all respondents recognized the potential value of diagnosing mTBI directly on the field and expressed unanimous interest in a POCT that uses a finger-prick blood sample for rapid assessment. This is especially pertinent given that venous blood sampling on the field is not feasible in 25% of cases.

While the heads of sports associations showed strong enthusiasm for POCT, they agreed that stopping a game to test an athlete would not be feasible. Instead, they proposed that injured athletes be taken out of play and tested afterwards to assess their readiness for future games or training. The game or race should proceed during this time.

Three key areas need attention to incorporate POCT into routine sports management. First is acquiring the necessary equipment to conduct these tests on-site (34%). Second, there must be a focus on training the staff to ensure they can perform these tests efficiently and accurately (33%). Third, the tests must be reliable and deliver accurate diagnostics to be helpful in sports (33%).

Leaders in sports associations display a comprehensive understanding of the profound implications associated with traumatic brain injuries. They are aware of the distinctions between mild and severe TBIs and the potential long-term effects of repeated injuries, such as hypopituitarism, encephalopathy, and other neurodegenerative diseases. They also recognize the importance of prompt medical intervention, which can significantly improve outcomes for injured athletes. Currently, POCT is not utilized on the field, but first responders or physicians commonly use standard medical devices for first aid and resuscitation at sports events. With adequate training, these professionals could also be equipped to administer POCT for TBI biomarkers, enhancing their ability to manage TBIs effectively during sports events.

## 4. Discussion

Implementing diagnostic processes encompasses interconnected aspects such as context analysis, identification of appropriate diagnostic tools, and characterization of diagnostic settings. Each element is pivotal in ensuring the efficacy and efficiency of medical diagnostics tailored to specific patient needs and diagnostic settings. By addressing these three areas, healthcare providers can develop a diagnostic strategy that is scientifically robust and practically viable. This holistic approach ensures that diagnostic processes are adapted to effectively address the specific health challenges and conditions of the population served. 

To implement biomarker testing in TBI management, according to the principle of value-based health care implementation, we performed a SWOT analysis, identifying strengths like improved diagnosis speed, weaknesses such as test costs, opportunities from POCT, and threats like diagnostic accuracy concerns. The analysis also identifies key stakeholders and diagnostic settings crucial for successful adoption.

Three diagnostic scenarios were developed, each with identified and classified key stakeholders. The scenarios include the analysis of TBI biomarkers using standard laboratory methods, POCT devices in the emergency room, and POCT devices used directly on-site. In Scenario 1, emergency department heads, neurologists, and traumatologists are the primary stakeholders. For scenarios 2 and 3, the same stakeholders are involved, with the addition of sports association heads to provide a broader perspective. By identifying stakeholders relevant to each scenario, the matrix ensures that feedback and insights come from the right professionals, enabling more informed decisions. This analysis is essential for understanding the requirements and challenges of implementing biomarker testing, with input from specialists refining guidelines to meet the needs of TBI management. 

Understanding the context in which these tests will be used is crucial to maximize their value. There are several crucial points that the laboratory and regulatory society must pursue regarding the possibility of using biomarkers in clinical practice. 

First, research and evaluations on many patients are needed, and large multicenter trials are required to define a practical guideline in association with the Italian Society of Emergency Medicine (SIMEU). A second issue is related to the evaluation of mTBI, which, if repeated, can cause cognitive degeneration, particularly affecting athletes and military personnel. There are also cases of secondary hypopituitarism that may be related to repeated mTBI, which may be relevant, especially in the sports setting.

Therefore, the optimal pathway for the patient with mTBI should provide the maximum care with the minimum waiting time. Introducing POCT can potentially streamline and enhance the management of TBI in emergency settings. Following the initial assessment, a biomarker test is administered, which can help quickly determine the severity of the injury and guide further treatment decisions without necessarily waiting for extensive laboratory results. Various POCT devices are used in ERs, highlighting a significant integration of these tests in practice. This utilization reflects a trend towards adopting more immediate diagnostic approaches that can lead to quicker clinical decisions, potentially reducing wait times and improving patient throughput. Such data are vital for understanding the penetration of advanced diagnostic technologies in emergency care and can serve as a baseline for further adoption and enhancement of POCT capabilities in healthcare settings.

TBI injuries, which can result in physical and psychological consequences, are worsened by misdiagnosis or lack of diagnosis. The Abbott Alinity mTBI test, in this context, can provide healthcare providers with an objective tool to help assess patients with suspected TBI and reduce their waiting time: a negative result can be used to rule out the need for a cranial CT scan, whereas a positive result could support clinicians in evaluating the trauma. The test would allow the quick exclusion of the need for a cranial CT scan in patients whose symptoms are not so acute, avoiding unnecessary observation times in the hospital and thus reducing overcrowding. 

The use of Abbott’s i-STAT Alinty TBI plasma test could therefore:

- have a significant impact on the time spent in the emergency department, reducing waiting time and shortening pre-treatment time (expected outcome: timeliness);
- improve the effectiveness of the whole TBI clinical pathway by reducing the time it takes to collect, process, and report results, with benefits for patients, clinical staff, hospital management teams, etc.;
- help provide targeted patient-centered care to improve patient care;
- reduce exposure to ionizing radiation for patients by improving prescribing appropriateness of computerized axial tomography;
- optimize sensitivity/specificity in identifying patients at greater risk;
- reduce the cost of the treatment.

There is already detailed information about pre-analytics and analytics. The very high negative predictive value, greater than 99%, is the strength of the Abbott Alinity mTBI test. 

To assess the impact and value of this new technology, we evaluated the actual capability of the markers to change the patient journey by simplifying it, reducing it, and making it more cost-effective. 

In addition, we included insights from interviews with healthcare professionals in emergency departments who expressed the practical advantages of biomarker testing. This section provides qualitative data on how implementing could alleviate operational challenges in high-pressure environments such as ERs.

An evaluation related to using different matrices regarding laboratory testing and POCT must now be carried forward. The next step will be the introduction of laboratory tests for evaluating TBI biomarkers in the ER, followed by assessing the expected value/benefits of tests in routine practice using key performance indicators. Therefore, in addition to the neurological and instrumental evaluation, patients who arrive at the ER with presumed TBI will be subjected to the analysis of TBI biomarkers, and the results of the laboratory test will be compared with the results of the CT scan. 

Biomarker testing offers numerous clinical benefits for patients, including reducing radiation exposure by decreasing the number of unnecessary CT scans, which is particularly beneficial for populations more vulnerable to radiation risks, such as children and young adults. Additionally, faster diagnosis and triage facilitated by biomarker testing can significantly reduce patient wait times in emergency departments, leading to quicker treatment initiation and a better overall patient experience. Finally, biomarkers provide critical information that enhances clinical decision-making, potentially leading to better patient outcomes through more accurate diagnosis and timely intervention.

The speed, portability, and high accuracy of the biomarker test would be especially relevant in military and sports settings, where TBIs are frequent and often underdiagnosed. In these contexts, an accurate and prompt diagnosis is even more critical to prevent second lesions before complete recovery from the first, reducing the risk of prolonged or permanent neurological damage. 

In sports medicine, the perception of TBI issues is positive, and the culture is present. Since an interruption of the game/match/race is not always possible, the best moment to perform the test would be at the end of the game/match/race to decide whether the athlete can continue training and competing in the following days or not, allowing an increase in safety and monitoring of the athletes, preventing return to play when the brain is still highly vulnerable to injuries. For this particular use, the development of a POCT test that works with a whole blood sample will be fundamental to facilitate the adoption of the test directly on the field, allowing the testing of athletes on-site immediately after the trauma, also contributing to reducing ER overcrowding and exposure to the ionizing radiation of the CT scan when not needed. The various sports settings differ, but a POCT test would be valuable and usable in all settings.

The first step in introducing biomarker testing in the sports context will be to test it on a sample of athletes to prove the efficacy and feasibility of biomarker use in the field. 

Large multicenter trials are needed to confirm the test’s value and introduce biomarker testing in federal protocols and guidelines. This highlights the importance of strategic investments in technology and training to facilitate the immediate and effective handling of traumatic brain injuries in sports environments, emphasizing that efficient diagnostics can substantially improve athlete safety and care.

## 5. Conclusions

The use of biomarkers aligns with value-based healthcare principles, emphasizing optimizing resource use, improving patient outcomes, and enhancing overall healthcare delivery efficiency. Biomarker testing has the potential to contribute to these goals by streamlining the diagnostic process, reducing unnecessary diagnostic imaging, and improving the allocation of medical resources.

The analysis of this case study shows that while this preliminary evaluation of biomarker potential is promising, further research is crucial to reassure clinicians about adopting these tests in emergency rooms. Similarly, trials in sports settings must integrate biomarker testing into federal protocols.

Comprehensive multicenter trials are needed inside and outside hospitals to assess the test’s utility across diverse contexts like sports, rural areas, and different age groups. Thus, developing a POCT test on capillary blood is fundamental to improving its effectiveness and easing the introduction of biomarker testing in emergency, sports, and military medicine contexts.

Laboratory specialists are pivotal in guiding the transformation process, offering their expertise to ensure effective implementation. Collaboration with device manufacturers will increasingly allow service quality to evolve across various therapeutic areas.

## Figures and Tables

**Table 1:** Graduated return-to-sport (RTS) strategy according to the 2017 Concussion in Sport Group (CISG) consensus statement.

Stage	Aim	Activity	Goal of Each Step
1	Symptom-limited activity	Daily activities that do not provoke symptoms	Gradual reintroduction of work/school activities
2	Light aerobic exercise	Walking or stationary cycling at slow to medium pace. No resistance training	Increase heart rate
3	Sport-specific exercise	Running or skating drills. No head impact activities	Add movement
4	Non-contact training drills	Harder training drills. E.g., passing drills. May start progressive resistance training	Exercise. Coordination and increased thinking
5	Full contact practice	Following medical clearance, participate in regular training activities	Restore confidence and assess functional skills by coaching staff
6	Return to sport	Normal game play	

Table from McCrory et al. Consensus statement on concussion in sport, the 5th international conference on concussion in sport held in Berlin, 2016. Br J Sports Med. 2017 Jun; 51(11):838–847 [24]

**Table 2:** Approaches and requirements for managing TBIs across different sports (summary of interview Sections S1 and S2 results for heads of sports associations).

Sport	
Rugby	TBIs are notably frequent, particularly during tackles. To manage this, matches require on-site medical personnel and ambulance services, and significant events necessitate alerting a neurosurgery center. Rugby also mandates online concussion management training for coaches and a minimum 30-day suspension from play following head injuries. Players must receive a negative CT scan and undergo motor rehabilitation before resuming training. Baseline neurological tests at the season’s start aid in managing injuries when they occur.
Boxing	Given the high incidence of TBIs, boxing matches are required to be held within an hour of a neurosurgery center, or a resuscitating physician must be present. The sport follows strict federal protocols, which include a mandatory precautionary suspension of 30 to 90 days post-injury. Boxers undergo medical evaluations before and after each match, and any head injury necessitates hospitalization and a CT scan.
Motorcycling	This sport includes various disciplines where races occur over extensive circuits equipped with medical stations and ambulances. Federal protocols dictate immediate medical intervention for any TBI occurrences. Pilots must receive clearance from a race medical officer to participate, and injuries result in being placed on an “unfit list”, barring them from future races until medically cleared.
Cycling	Races are supported by ambulances and medical cars, with several medical stations available along the routes. Trauma management is left to the discretion of attending physicians, and the federation does not demand post-injury medical assessments beyond the required annual medical certification.
Sailing	TBIs are rare in sailing, making widespread testing less applicable. However, the potential for POCT performed by non-medical staff is being considered for introduction at sailing clubs, accommodating the sport’s specific needs.

## Data Availability

No new data were created or analyzed in this study. Data sharing is not applicable to this article.

## Supplementary Materials

The following supporting information can be downloaded at: https://www.mdpi.com/article/10.3390/jpm14060634/s1.
