# Supplementary material for: Value-Based Health Care Implementation: The Case Study of mTBI Biomarkers
Source: J Pers Med. 2024 Jun 14;14(6):634. doi: 10.3390/jpm14060634 (PMC11204511; doi:10.3390/jpm14060634)
Supplement: Supplementary file 1 [file jpm-14-00634-s001.zip › jpm-3036460-supplementary.pdf]

Annex 1

INTERVIEW FOR HEAD ER PHYSICIANS

OBJECTIVES AND METHODOLOGY

Investigation on the current management of subjects with suspected TBI in order to evaluate the introduction of a test for biomarkers of TBI. The latter have the potential to improve patient management and optimize the use of resources in the emergency department, improving the diagnostic accuracy and reducing the number of unnecessary CT scans performed on patients with suspected TBI.

The interview consists of three sections with questions related to 1) management and facility, 2) laboratory tests, and 3) biomarkers of TBI.

Identification data:

Name:______________________________ Surname:__________________________________

Center:____

Date:______________________

Section 1: QUESTIONS RELATED TO THE FACILITY AND TAKING IN CHARGE

1. **What standard guidelines or recommendations do you currently refer to?**
    - Canadian CT Head Rule
    - Scandinavian Guidelines
    - New Orleans Criteria
    - Guidelines of the National Institute for Health and Care Excellence (NICE 2014)
    - Neurotraumatology Committee of the World Federation of Neurosurgical Societies (NCWFS)
    - Other (specify): ____
    - None

2. **Describe the order of events that occurr since the patient arrives at the emergency department (number):**
    - Access to the emergency room
    - Triage
    - Specialist consultation
    - Tac
    - Laboratory tests

- o Hospitalization/Discharge
- o Other (describe) ____

3. **How much time elapses between access to the emergency department and the decision for the Admission/Discharge?**
   With positive CT scan ____
   With negative CT scan ____
   Other____

4. **Who is involved in the took in charge of a patient with TBI from the time of arrival at the emergency department to the time of discharge?**
   - o Medical staff
   - o Nursing staff
   - o Staff for CT scans
   - o Other (specify): ____

5. **Who is involved in the <u>treatment</u> of a patient with TBI from the time of arrival in the emergency department to the time of discharge?**
   - o Medical staff
   - o Nursing staff
   - o Staff for CT scans
   - o Other (specify): ____

6. **Which elements are currently taken into account at the time of admission? (e.g. GCS,)**
   - o CGS
   - o Age
   - o Anticoagulant therapy
   - o Risk Factors
   - o Trauma dynamics
   - o Other (specify): ____

7. **Is CT scan in the emergency room?**
   - o YES

- NO

8. **If yes, is it available quickly (within 2 hours from the arrival at the ER)?**
   - YES
   - NO

9. **If yes, available in less than 1 hour?**
   - YES
   - NO

10. **If yes, available in less than 2 hours?**
    - YES
    - NO

11. **If so yes available in less than 3 hours?**
    - YES
    - NO

12. **If yes, available in over 4 hours?**
    - YES
    - NO

13. **If yes, is the staff (technicians) present 24/24h?**
    - YES
    - NO
    - On call

14. **If yes, is the staff (radiologists) present 24/24h?**
    - YES
    - NO
    - On call

15. **Would it be useful to be able to rule out the need to perform a CT scan directly upon arrival at the emergency room?**

- YES
- NO

Section 2: QUESTIONS RELATED TO LABORATORY TESTING

16. **Is there a laboratory for analysis in your facility?**
    - YES, and it's only for emergencies
    - YES, and it's centralized
    - NO

17. **If yes, do you routinely request laboratory tests?**
    - YES
    - NO

18. **If yes, does it respond effectively to the needs of the emergency department?**
    - YES
    - NO

19. **If yes, is it available 24/24h?**
    - YES
    - NO

20. **Are laboratory tests (clinical biochemistry, blood count, coagulation, ...) usually required in case of TBI?**
    - YES
    - NO

21. **If not, is it because you think that the laboratory analysis do not give any additional value to the CT scan information?**
    - YES
    - NO

22. **If not, is it because you think that laboratory analysis cannot improve the diagnostic pathway?**

- YES
- NO

23. If not, is it because they are not available?

- YES
- NO

24. If yes, which ones?

- Hematology routine
- Coagulation
- Blood count
- Hepatic/renal function
- D-dimer
- Cardiac enzymes
- Other (specify): ____

25. If yes, are they required for diagnostic purposes?

- YES
- NO

26. If yes, are they required for prognostic purposes?

- YES
- NO

27. If yes, how long does it take to get the results during the day?

- Less than 1 hour
- More than 1 hour
- More than 2 hours

28. If yes, how long does it take to get the results at night?

- Less than 1 hour
- More than 1 hour
- More than 2 hours

29. If yes, do you think these tests improve the diagnostic pathway?

- YES
- NO

30. If yes, why do you think these tests improve the diagnostic pathway?

1. Because they detect coagulation abnormalities
2. Because they detect changes in the blood count
3. Because they allow to frame the patient's general state of health
4. Because they allow a diagnosis about the reason for the trauma
5. Other (specify): ____

31. If yes, are they intended to reduce the number of performed CT scans?

- YES
- NO

Section 3: QUESTIONS RELATED TO BIOMARKERS OF TRAUMATIC BRAIN INJURY

32. Do you know any TBI biomarkers?

- YES
- NO

33. Would you adopt TBI biomarkers assessment in your emergency department?

- YES
- NO

34. In particular, are you familiar with GFAP and UCH-L1 biomarkers?

- YES
- NO

35. If yes, do you know the diagnostic windows of GFAP and UCH-L1 biomarkers?

- YES
- NO

36. If yes, do you think that GFAP and UCH-L1 biomarkers may have a utility in identifying mild TBIs?

- YES
- NO

37. Would you adopt GFAP and UCH-L1 biomarker assessment in your emergency department?

- YES
- NO

38. If yes, would you adopt them as laboratory tests or as POCT?

- Laboratory Tests
- POCT
- Both

39. If yes, would you consider it useful to include the test in the routine in the emergency room for the purposes of:

- Diagnosis
- Prognosis
- Therapy
- Monitoring
- Screening
- Patient management (reduction of observation time)

40. If yes, would it be helpful for reducing the observation time for patients in the emergency department?

- YES as POCT
- YES as a laboratory test
- NO as POCT
- NO as a laboratory test

41. If yes, would it be useful to assign the urgency code?

- YES as POCT
- YES as a laboratory test

- NO as POCT
- NO as a laboratory test

42. If yes, when would you consider useful to perform the test?

- At the time of triage
- After Acceptance
- Before the CT scan
- After the CT scan

43. If yes, in which age groups would you consider it appropriate to perform the test:

- 0-16
- 17-59
- 60-74
- >75

44. If yes, would you consider it appropriate to perform the test for diagnostic or prognostic purposes?

- Diagnostic
- Prognostic

45. If yes, would you perform the test in exclusion of the first CT scan, or would you prefer to keep both?

- Biomarkers testing only
- CT scan only
- Biomarkers test & CT Scans

46. If yes, would you perform the test in exclusion of a second CT scan, or would you prefer to keep both?

- Biomarkers testing only
- CT scan only
- Biomarkers test & CT Scans

47. **If yes, are there any age groups in which you would still consider it necessary to perform a CT scan?**
   - o 0-16
   - o 17-59
   - o 60-74
   - o >75 mm

48. **If yes, which are the conditions necessary for the inclusion of the test as a POCT in the routine in the emergency room?**
   - o Presence of laboratory instrumentation
   - o Properly trained staff
   - o Good diagnostic accuracy
   - o Other (specify) ____

49. **If biomarker assessment was available on capillary blood in a matter of minutes, would you use it as a POCT?**
   - o YES
   - o NO

50. **If yes, would you have staff to administer the test?**
   - o YES
   - o NO

51. **If the test could be performed as a POCT at the site of the trauma, would you still repeat the laboratory test in the emergency room?**
   - o Only on the field
   - o On the field and in the emergency room
   - o Emergency room only

52. **If the test could be performed as a POCT in the emergency room, without the need to process the samples in the laboratory, would you still asks for a confirmatory laboratory test for the same markers?**
   - o YES

- NO

53. Do you routinely use POCTs in your emergency room?

- YES
- NO
- I don't know

54. If yes, which ones?

____

55. Do you usually request a validation of the results through laboratory analysis in the case of a POCT carried out in the emergency room?

- YES
- NO

56. Do you usually request a validation of the results <u>through laboratory analysis</u> in the case of a POCT carried out outside the emergency room?

- YES
- NO

57. Are you used to <u>repeat</u> in the emergency room a <u>POCT</u> already carried out outside the emergency room?

- YES
- NO

58. What parameters would you consider when comparing two different diagnostic tools (two laboratory tests, tests vs POCT, ...)?

- Cost of the test
- Time required to perform the test
- Need for laboratory instrumentation
- Need to train staff
- Required manpower and work
- Consumption of reagents for execution
- Specificity

- Sensibility
- Accuracy
- Number of CT scans saved
- Test Validation
- HTA
- Other (specify) ____

59. Which aspects do you consider most important in the patient journey?

- Fast diagnosis
- Accuracy of diagnosis
- Need for hospitalization (rule in/out)
- Number of CT scans performed
- Time spent in the emergency department
- Other (specify) ____

60. Would you consider it useful to perform the test in young patients in a sports context?

- YES
- NO

61. If yes, in which age groups?

- 0-16
- 17-59
- 60-74
- >75 mm

Annex 2

INTERVIEW FOR SPORTS ASSOCIATIONS

OBJECTIVES AND METHODOLOGY

Investigation on the management of athletes with suspected TBI in order to evaluate the introduction of TBI biomarker testing to offer timely diagnosis and allow appropriate assessments before the athletes return to play/training.

The interview is composed of three sections with questions related to 1) the sports center, 2) trauma management, and 3) testing and biomarkers.

The interview is addressed to the directors of sport-associations and federal physicians.

Identification data:

Name: ______________________________ Surname: _________________________________

Center:____

Date:______________________

Section 1: QUESTIONS RELATED TO THE SPORTS CENTER

1. **Indicate the type of sport practiced**:

   ____

Section 2: QUESTIONS RELATED TO TRAUMA MANAGEMENT

2. **Are there currently standard guidelines or recommendations in use for the management of head trauma?**
   - o YES
   - o NO

3. **If so, which ones?**

   ____

4. **Is emergency services always required in the event of a blow to the head?**
   - o YES

- NO

5. **<u>Who</u> is responsible for assessing a potential head injury at the time of the accident to decide whether to call for help?**
    - Coach
    - Infirmary
    - Field Medic
    - Company Personnel
    - Other socio-economic impacts: ___

6. **<u>How</u> is a head injury assessed (based on what parameters or assessments) at the time of the accident to decide whether to call for help?**
    - Verbal response to a certain vocal stimulus
    - Motor response to a given command
    - Opening the eyes
    - Loss of consciousness
    - Amnesia
    - Confusion
    - Seizures
    - Vomit
    - Headache
    - Signs of bruise/fracture
    - Other socio-economic impacts ___

7. **How long does it take for help to arrive?**
    - 15 min
    - 30 min
    - 45 min
    - 1 h
    - More than 1 h

8. **Indicate the subjects involved in the management of athletes with a suspected head injury from the time of trauma to arrival in the emergency department:**

- Coach
- Infirmary
- Field Medic
- Company Personnel
- Other socio-economic impacts ___

9. How many staff you have on the sidelines would be able to handle a head injury?

____

10. What kind of courses did they take?

____

11. How far away (km) is the emergency room from the facility where sports are practiced?

____

12. How long does it take to get to the emergency room from the facility where you play sports?

____

13. Generally, how long after discharge from injury does the athlete resume training?

____

---

14. Are diagnostic tests normally required before resuming training?

- YES
- NO

15. If so, which ones?

____

Section 3: QUESTIONS RELATED TO TESTING AND BIOMARKERS

16. Do you know about TBI biomarkers?

- YES

- NO

17. Are you particularly familiar with GFAP and UCH-L1 biomarkers?

- YES
- NO

18. Would you think it would be useful to be able to identify a minor head injury directly at the scene of the accident?

- YES

  If so, why?__
- NO

  If not, why?__

19. Would you consider it useful to be able to identify a head injury directly at the time of the injury on the basis of biomarkers?

- YES
- NO

20. Would anyone be able to take a venous blood sample directly in the field for this purpose?

- YES
- NO

21. Would you consider useful a device that evaluates biomarkers through a drop of blood obtained by finger prick directly on the sidelines?

- YES

  If so, why?__
- NO

  If not, why?__

22. Do you know the difference between severe head trauma and mild head trauma?

- YES
- NO

23. **Are you aware that head trauma, if repeated, can lead to serious pathologies including acquired hypopituitarism, chronic traumatic encephalopathy and other neurodegenerative diseases?**
    - o YES
    - o NO

24. **Are you aware that the prognosis of a head injury depends on the timeliness of the intervention?**
    - o YES
    - o NO
